# Editorial

**Published:** 1891-12

**Authors:** 


### Editorial.

### MEDICINE IN GEORGIA.

The status of medical affairs in Georgia—her laws, ethics, professional requirements, etc.—is certainly in no favorable condition. Not only is it the hotbed of the patent medicine fiend, but quacks and charlatans flourish like the "green bay tree." Medical advertising is evidently the leading question, and many vie with each other as to who can do it in the most delicate manner. Every question has two sides, and there are a goodly number of clear-minded men who advocate that there is a difference between the present code of medical ethics and that of our more ancient colleagues, made necessary, they aver, by the progress of the times. Others, indeed, "our chivalric fathers," the truest types of the medical profession, believe that no period of time can efface those professional principles, ethics, let us say, cherished and possessed by every right thinking Christian physician. The former class believe that this is the age for show, the age of competition, and for them to succeed, they must display before the eyes of the public some wonderful healing power possessed by none of those claiming the distinction as physicians. Surely we are returning to the midæval ages, where witchcraft soars above knowledge and reason. But truth and rectitude will appeal to the conscience of every man who has those principles dwelling in him. Some things can be proven by argument, and yet the grandest truths are those which need no proof, but whose very existence is stamped upon their face. Does any human being in whom there is any quality of his Divine Maker need proof that a cold-blooded murder is wrong? So it is with medical ethics. While we may not think that the code as formulated by our fathers is in every respect suited to the present times, yet they are principles which have so long governed us, and principles so fully received by the finer instincts of our nature, nor are they obsolete, that no fair-minded Christian physician would have these principles of our ethics relegated to the past, and be substituted by such as would be formed by those knifeless chirurgyists of the nineteenth century. Environments cause changes; finer technique in experimentation may place us beyond the age of Hippocrates, but no minds of our present day can eclipse the subtle reasoning of our past men of science. And why should we of the present day wish to change those principles of medical ethics which, deep down in our consciences, we believe to be right? Are not the same self-evident truths of a hundred years ago present with us to-day? True worth can never be "hid under a bushel." The professional man of ability needs no flaming advertisement to be placed before the eyes of the public, for still water always runs deep. Nor is it a barbarism of an obsolete age. The South, phœnix-like, is leaving behind her the smouldering ashes of credulity, and, like as is her rise in the commercial world, so is her knowledge for discriminating between true worth and shams. Let our professional brethren bear in mind that we are not practicing our profession for notoriety, but let all consider their work as governed by a still nobler professional principle.

Had Georgia a more efficient medical law, the state of affairs would be far different, for as it is, every man under the title of doctor comes to this State after he has failed to be allowed to practice in any other, because he knows that he has only to show something like a diploma, and often nothing, depending simply upon the man's oath, after which he is allowed to inflict his barbarities upon an unsuspecting public. Thus the physician who has expended years and money upon his medical education must contend with such a class. The dignity of the medical profession will sink lower and lower unless physicians firm of integrity will stand shoulder to shoulder and fight for a standard on which it should rest. The public will never do it, for until they become educated to it, that had just as soon have an itinerant doctor as a reputable physician, provided he will do the work a dollar cheaper.

We appeal to the physicians of our State to rest awhile and consider the status of affairs. Our State medical laws are lax, and for the good of the public and ourselves we should be more vigilant in aiding the enactment of that section of our code which says: "It is the duty of physicians who are frequently witnesses of the enormities committed by quackery, and the injury to health, and even destruction of life caused by the use of quack medicines, to enlighten the public on these subjects, to expose the injuries sustained by the unwary from the devices and pretensions of artful empirics and impostors." Many a promising young physician is led to flaming advertisement, and then to quackery, by the fact of his eyes being dazzled by a possible quick way of gaining the filthy lucre, and thus losing sight of the nobler idea of our profession. The road to distinction and success is no rapid transit; but true worth and integrity in the end will far outstrip this "sounding brass and tinkling cymbal," and give to all a name far more imperishable, to be loved and cherished by those whom you served.

### DR. J. McFADDEN GASTON.

We are glad to know that Dr. J. McFadden Gaston, of Atlanta, was elected president of the Southern Surgical and Gynecological Society, at its last meeting in Richmond, Virginia. To be elected president of such an active, working society, numbering, as it does, so many of the leading lights of the medical profession, and accomplishing such proficient work, is an honor of which one might well be envious. By the election of Dr. Gaston the profession of Atlanta may feel complimented, and that it is an honor well bestowed.

WE note with pleasure the appearance of an article by Dr. A. G. Hobbs, of this city, in the last issue of the *Journal of Laryngology and Phrenology*, published in London. "The Voice and its Treatment," is the title, and admirably handled, as would naturally be expected, from the pen of one of such vast experience in this line of work.

*The New York Journal of Gynecology and Obstetrics.* Issued monthly. Edited by A. H. Buckmaster, M. D., and J. D Emmet, M. D.

The November number of this journal, which is the first number of volume first, is now before us. "Our work," says the editor, "is designed to give to the medical profession a monthly journal of the highest grade in all its departments.' Thus far they have hewn to the mark, and we believe this journal is destined to become indispensable to the general practitioner. We are pleased to place it on our list of exchanges.

---

*The Sanitarium.* A Journal of Preventive Medicine and Hygiene. Published monthly at Austin, Texas, by the Sanitarium Publishing Company. T. J. Bennett, Managing Editor. Two dollars a year, twenty cents a copy

The first number of this attractive journal has just been received, and we place it on our exchange list with some degree of pride, believing that it is destined to a long and useful life. This journal fills a long felt want in the South. To be accomplished and useful practitioners, a knowledge of causes and the proper methods of preventing disease are essential We take pleasure in recommending this journal to the practitioner

---

### LAVAGE OF THE STOMACH IN YOUNG CHILDREN

(Wratch, No 26 and 27, 1890.) Froitzky publishes his results in the treatment of a series of sixty-four cases of gastro-intestinal diseases by this method. The age of the children varied from two weeks to two months. On the average, two lavages were required to control vomiting. The operation is readily performed, and is well borne by even the youngest child; the only apparatus being a Nelaton tube and a glass funnel. The author uses a solution of boiled water containing three per cent of salicylate of soda. The most favorable results are obtained in dyspepsia, without fever, especially when the stomach alone is at fault; cure is less rapid in gastro-intestinal affections; and still less rapid when the disease is confined to the intestinal canal. In acute gastro-intestinal disease, including the specific summer diarrhœas, lavage is useful, but does not suffice to the exclusion of other therapeutic measures. Chronic gastro-intestinal diseases are benefited by lavages employed in conjunction with other means indicated by the condition of the patient.—*Annals Gyn.—Toledo Med. Compend.*

